# Supplementary material for: Barriers and Enablers for Equitable Healthy Food Access in Baltimore Carryout Restaurants: A Qualitative Study in Healthy Food Priority Areas
Source: Nutrients. 2024 Sep 8;16(17):3028. doi: 10.3390/nu16173028 (PMC11396806; doi:10.3390/nu16173028)
Supplement: Supplementary file 1 [file nutrients-16-03028-s001.zip › nutrients-3198103-supplementary.pdf]

Complex carryout food systems: Participatory modeling to examine inequalities in access to healthy prepared food in Baltimore

PI Name: Yeeli Mui
Study Title: Complex carryout food systems: Participatory modeling to examine inequalities in access to healthy prepared food in Baltimore
IRB No.: IRB00022449
PI Version No. / Date: Ver 2 - 11/3/2022

IN-DEPTH INTERVIEW GUIDE

Carryout Owner

Greeting: Hello, I am ___________ from the Johns Hopkins Bloomberg School of Public Health and I wanted to thank you for being willing to speak with us today about your experience as a carryout owner.

Part 1. General. I will begin with a couple of general questions to get to know you

1. Tell me a little bit about yourself and your carryout restaurant

   Potential probes:

   - *Tell me the story about how this carryout got started…*
     1. *How has your carryout changed over time? (demands, offerings, etc)*

Part 2. Operations of the carryout. We're going to move on to talk more about a typical day in your Carryout and how you run your business.

2. Can you describe your day-to-day Carryout operations?

   Potential probes:
   - *Who are the people you are serving?*
   - *What are your hours of operation?*
   - *Probe somewhere on online ordering, take out business, etc. how that all works…*

3. Tell me about who else works with you at the carryout and their roles.

   Potential probes:
   - *How do you train your staff?*

4. How do you decide what to offer on your menu?

   Potential probes:

- *Where do the recipes you follow come from?*
- *What factors influence the foods you offer?*
- *Tell about how you make changes to your menu*

I don't see much on things directly related to the FRESH intervention – ie how food is prepared, training of staff/cooks, types of promotions that work with customers…

Part 3. Food shopping The next set of questions will ask about your experiences obtaining food for your restaurant.

5. Please describe how you purchase the food for your carryout:

   Potential probes:

   - *Who are your top 2-3suppliers (i.e. where do you buy from most)?*
   - *Tell me about the delivery options you have*
   - *How often do you go to a supplier or store to buy needed ingredients?*
   - *Tell me about any specific specialty category of items you seek out such as organic, local, etc. and where you buy it*

6. If you could change how you buy/receive your food, what would that look like?

Part 4. Relationships. We are going to move onto questions about your relationship with customers and others that support your business.

7. Tell me about your customers

   Potential probes:

   - *Who are the people you serve?*
   - *How do customers give you feedback?*

8. What do you see your carryout bringing to the community?
9. What other relationship do you rely on for a successful business?

   - *Other carryout owners*
   - *Non-profits*
   - *Government*

- *Other food sector workers*

Part 5. Food Policies We're almost done, but want to talk about what would help your business.

10. Thinking about these different topics we've discussed, what are the major challenges that you have as a carryout owner and what has been successful?

11. If you could talk to a policy maker what would you like them to know and what would you like them to do for you?

12. What sorts of changes would you like to see (at different levels) for your restaurant?
